# Integrin α2 is an early marker for osteoclast differentiation that contributes to key steps in osteoclastogenesis

**DOI:** 10.3389/fcell.2024.1448725

**Published:** 2024-08-15

**Authors:** Katrin Brockhaus, Isabel Hemsen, Saskia-Larissa Jauch-Speer, Stephan Niland, Thomas Vogl, Johannes A. Eble

**Affiliations:** ^1^ Institute of Physiological Chemistry and Pathobiochemistry, University of Münster, Münster, Germany; ^2^ Institute of Immunology, University of Münster, Münster, Germany

**Keywords:** osteoclast, integrin α2β1, CD9, collagen, differentiation

## Abstract

**Introduction:**

Osteoclasts determine bone tissue turnover. Their increased activity causes osteoporosis, their dysfunction osteopetrosis.

**Methods and Results:**

Murine monocytic ER-Hoxb8 cells differentiate into OCs upon treatment with M-CSF and RANKL and upregulate the collagen-binding integrin α2β1 distinctly earlier than other OC markers, such as the OC-associated receptor, OSCAR. Integrin α2β1 promotes OC differentiation at multiple levels by stimulating differentiation-relevant genes, by regulating cell matrix adhesion and the formation of adhesion-promoting protrusions, and by the upregulation of proteins involved in precursor cell fusion. The two key factors in osteoclastogenesis, RANK and NFATc1, were essentially unaffected after knocking out the ITGA2 gene encoding integrin α2 subunit. However, compared to integrin α2β1 expressing ER-Hoxb8 cells, ITGA2-deficient cells adhered differently with more branched filopodia and significantly longer tunneling nanotubes. Despite the higher number of fusion-relevant TNTs, they form fewer syncytia. They also resorb less hydroxyapatite, because integrin α2β1 regulates expression of lacuna proteins necessary for bone matrix resorption. The impaired syncytia formation of ITGA2-deficient OC precursor cells also correlated with reduced gene activation of fusion-supporting DC-STAMP and with an almost abolished transcription of tetraspanin CD9. CD9 only partially colocalized with integrin α2β1 in TNTs and filopodia of integrin α2β1-expressing OC precursors.

**Discussion:**

Our findings define integrin α2β1 as an early marker of OC differentiation.

## 1 Introduction

Osteoclasts (OCs) are multinucleated cells that arise from the fusion of mononuclear hematopoietic precursor cells of the monocyte/macrophage lineage (Boyle et al., 2003; Amarasekara et al., 2018). They mediate bone resorption and, together with the bone forming osteoblasts, orchestrate bone homeostasis (Boyle et al., 2003). Since their increased activity causes osteoporosis and their dysfunction osteopetrosis, a better understanding of osteoclast differentiation and the regulation of their bone matrix resorption activity is highly desirable. The differentiation of OCs, termed osteoclastogenesis, is mainly regulated by two cytokines, macrophage-colony stimulating factor (M-CSF) and receptor activator of NF-κB ligand (RANKL). Initiating osteoclastogenesis, M-CSF induces monocytic OC-precursor cells to express the receptor activator of NF-κB (RANK). Via RANK, OC-precursors are activated by RANK ligand (RANKL) and thus progress differentiation by expressing specific genes (Amarasekara et al., 2018), among which is the nuclear factor of activated T-cells 1 (NFATc1), the master regulator of osteoclastogenesis. This transcription factor leads to expression of OC markers, such as tartrate-resistant acid phosphatase (TRAP), dendritic cell-specific transmembrane protein (DC-STAMP), the collagen-binding osteoclast associated receptor (OSCAR), and cathepsin K (Asagiri and Takayanagi, 2007).

Integrins are heterodimeric receptors mainly mediating cell-matrix interactions. Attaching to bone matrix, OCs form a sealing zone composed of densely arrayed podosomes. Their core consists of F-actin and actin-associated proteins, which is surrounded by a ring-like zone of integrins and integrin-associated proteins (Georgess et al., 2014; Soysa and Alles, 2016). Furthermore, integrins convey extracellular signals that presumably regulate OC differentiation (Duong et al., 2000; Zou and Teitelbaum, 2010; Lu et al., 2016). The main integrin expressed by bone resorbing OCs is integrin αVβ3, which mediates initial adhesion of OCs to its extracellular matrix ligands that contain RGD sequences, including vitronectin, osteopontin, bone sialoprotein II and a cryptic RGD-site in denatured collagen type I (Nesbitt et al., 1993; Duong et al., 2000). Although αVβ3 promotes OC migration, survival, formation of the sealing ring, and bone resorption (Nakamura et al., 2007), it is dispensable for osteoclastogenesis *in vivo* (Chambers and Fuller, 2011), likely due to a compensatory upregulation of the collagen-I binding integrin α2β1 (Horton et al., 2003; Chambers and Fuller, 2011). Collagen-I is the most abundant organic component of the bone matrix and highly relevant for integrin α2β1 mediated OC adhesion. Integrin α2β1 colocalizes with F-actin in structures that are relevant for bone resorption: podosomes (Song et al., 2014) and the ruffled border protruding into the lacuna of OCs (Helfrich et al., 1996). Integrin α2-and β1 antibodies reduce osteoclastic bone resorption (Duong et al., 2000). Furthermore, integrin α2β1 enables OC precursor cells to migrate and to approach each other, a prerequisite to fuse into multinucleated OCs (Helfrich et al., 1996; Rucci and Teti, 2016). For a subset of monocytic cells, integrin αMβ2 also promotes adhesion to the bone surface (Sprangers et al., 2017).

Primary OCs are difficult to isolate due to their size and fragile morphology, making them unsuitable for knockout and similar studies. Therefore, to study the role of integrin α2β1, we employed the murine cell line ER-Hoxb8 as *in vitro* model of OC differentiation. ER-Hoxb8 cells are conditionally immortalized murine monocytic precursor cells that carry the class I homeodomain transcription factor Hoxb8 fused to the estrogen-binding domain of the estrogen receptor (Wang et al., 2006; Di Ceglie et al., 2017). We show that the integrin α2 subunit-encoding gene is transiently expressed at the onset of osteoclastogenesis, peaking around day 2. Integrin α2β1 stimulates the expression of several genes involved in the formation of cell protrusions, cell fusion and degradation of the bone matrix in the lacuna, and thus, although not indispensable, contributes significantly to OC differentiation.

## 2 Materials and methods

### 2.1 Culture and differentiation of ER-Hoxb8

ER-Hoxb8 cells (originally provided by Prof. Dr. H. Häcker, St. Jude Children’s Research Hospital, Memphis, TN, United States) (Wang et al., 2006) were cultured in 6-well plates in RPMI 1640 (Sigma-Aldrich, St.Louis, MO, United States) containing 10% FCS (Capricorn Scientific, Ebsdorfergrund, Germany), 2 mM L-glutamine, 100 I.U. penicillin, 10 μg/mL streptomycin, 1 µM estradiol (all from Sigma Aldrich, St.Louis, MO, United States) and 1% conditioned medium from GM-CSF producing B16 melanoma cells. For differentiation into OCs, ER-Hoxb8 cells were washed with PBS +10% FCS to remove estradiol, seeded into 24-well plates at a density of 125,000 cells/well in 1 mL α-MEM (Lonza, Basel, Switzerland) containing 5% FCS, 2 mM L-glutamine, penicillin/streptomycin, 0.5 mM phospho-ascorbic acid (Sigma-Aldrich, St.Louis, MO, United States), 30 ng/ml M-CSF (Peprotech, Cranbury, NJ, United States) and 20 ng/mL RANKL (Bio-Techne, Minneapolis, MN, United States), and incubated in a humidified incubator at 37°C and 5% CO_2_.

### 2.2 RNA isolation and quantitative real-time PCR

Total RNA of cells was isolated using RNeasy Mini Kit (Qiagen, Hilden, Germany) and reverse transcribed into cDNA using the Reverse Transcriptase Core Kit (Eurogentec, Seraing, Belgium). Quantitative real-time PCR was performed using the QuantiNova SYBR Green PCR Kit (Qiagen, Hilden, Germany) and the Rotor-Gene Q system (Qiagen, Hilden, Germany) according to the manufacturer’s protocol. The gene-specific primers for transcript quantification are listed in Supplementary Table S1. Gene expression was analyzed using the ∆∆Ct method with GAPDH as control.

### 2.3 CRISPR/Cas9 knockout of ITGA2

ITGA2 knockout of ER-Hoxb8 cells was performed by lentiviral CRISPR/Cas9 transduction. To this end, a gRNA sequence corresponding to exon 3 of murine ITGA2 gene was designed using idt SciTools Web Tool (integrated DNA technologies, Leuven, Belgium; https://eu.idtdna.com/pages/tools) avoiding any off-targets. The respective guide oligonucleotides with BsmBI overhangs (forward 5′-CAC​CGC​TGG​TTG​GTT​CAC​CGT​GGA​G-3′, reverse 5′-AAA​CCT​CCA​CGG​TGA​ACC​AAC​CAG​C-3′) were cloned into the BsmBI-site of lentiCRIS-PRv2 (Plasmid #52961 Addgene, Watertown, MA, United States). After verification of the gRNA-insert by sequencing, lentiviruses were produced by co-transfection of HEK293FT-cells with this lentiCRIPSRv2-ITGA2gRNA construct, the packaging plasmid psPAX2 (Addgene #12260) and the envelope plasmid pMD2.G (Addgene #12259), and then transduced into ER-Hoxb8 cells. Puromycin (InvivoGen, San Diego, CA, United States) at 8 μg/mL was used for selection. Subclones from single cells were checked by T7 endonuclease assay, regular PCR and sequencing.

### 2.4 Confirmation of ITGA2 knockout by T7-endonuclease test and sequencing

ITGA2 knockout was verified with primer pairs flanking the target site in exon 3 of integrin α2 (α2g1Cas forward 5′-GTC​CTG​GTC​AGC​TAT​GAT​GAC-3′, α2g1Cas reverse 5′-TAA​CAG​GGG​TGT​GCC​TAC​AC-3′). Genomic DNA of ER-Hoxb8 cells and CRISPR/Cas9-treated cell clones was isolated using the QIAamp DNA Mini Kit (Qiagen #51304). PCR was performed with 100 ng genomic DNA, 10 µM of each primer and Taq DNA polymerase, and PCR products were purified with the Zymo Clean and Concentrator Kit 5 (Zymo Research, Freiburg, Germany). 200 ng of purified PCR products were incubated with T7 endonuclease I (New England Biolabs, Ipswich, MA, United States) according the manufacturer’s instructions. Genomic indels were detected by agarose gel electrophoresis and the efficiency of gene modification was calculated according to Guschin et al. (2010) as ca. 12%, which is likely underestimated (Guschin et al., 2010), but in the normal efficiency range (Konstantakos et al., 2022).

In addition, the integrin α2 knockout was confirmed by sequencing. For this purpose, clones were isolated from the ITGA2-KO cell population. From their genomic DNA, the relevant gene sequence was amplified by PCR with the high-fidelity KAPA HiFi DNA-polymerase (Roche Diagnostics, Mannheim, Germany) and the α2g1Cas forward and reverse primers, TOPO-cloned in pCR2.1-TOPO (Thermo Fisher Scientific, Darmstadt, Germany) and sequenced with M13rev-29 primer (5′-CAG​GAA​ACA​GCT​ATG​ACC-3’; Eurofins Genomics, Ebersberg, Germany). By sequencing, 11 out of 12 clones were proven to be negative for both ITGA2 alleles.

### 2.5 Magnetic bead-based cell sorting for M-CSF receptor and flow cytometry

ER-Hoxb8 ITGA2-wildtype (ITGA2wt) and integrin α2 knockout (ITGA2-KO) cells with high M-CSF receptor expression were enriched twice with the CD115 MicroBead Kit (Miltenyi Biotec, Bergisch-Gladbach, Germany) according to the manufacturer’s instructions. Enrichment was quantified by flow cytometry. To this end, 1×10^6^ cells were resuspended in cold MicroBead sorting buffer, blocked with FcR blocking reagent, and stained with phycoerythrin (PE)-conjugated CD115 antibodies (human anti-mouse, PE, REAlease, #130-118-071, Miltenyi Biotec) or control antibody (human IgG1, PE, REAfinity, #130-113-450).

### 2.6 Histological staining of OCs for TRAP-activity

ER-Hoxb8 cells were differentiated on uncoated and collagen-I-coated glass coverslips for 7 days, washed with PBS, fixed with 2% PFA and permeabilized with 0.1% Triton-X100. Subsequently, they were stained for TRAP activity using the leukocyte acid phosphatase kit (Sigma-Aldrich, St. Louis, MO, United States), according to the manufacturer’s protocol. After washing with deionized water and embedded with fluorescent mounting medium (Agilent, Santa Clara, CA, United States), samples were imaged using a confocal laser scanning microscope (LSM 800, Zeiss, Wetzlar, Germany).

### 2.7 Immunofluorescence and nuclear staining

For nuclear staining and immunofluorescence, cells were fixed with 2% PFA in PBS for 10 min and washed thoroughly with PBS. After permeabilization with 0.1% Triton-X100 or 0.1% saponin in PBS, samples were blocked with 5% normal serum matching the secondary antibody and 1% BSA in PBS for 1 h. Subsequently, they were incubated overnight at 4°C with primary antibodies against integrin α2 (1:500, monoclonal rabbit NBP2-67691, Bio-Techne, Abington, United Kingdom), integrin αV (1:100, polyclonal rabbit antibodies, Bioss bs-1310R, Woburn, MA, United States), DC-STAMP (1:50, monoclonal mouse MABF39-I clone 1A2, Sigma-Aldrich), and CD9 (1:500, monoclonal rat MA1-10309, Invitrogen, Waltham, MA, United States) in blocking solution. After washing with PBS, samples were incubated with fluorescently labeled secondary antibodies, donkey anti-rabbit Alexa Fluor 488 (Invitrogen A21206), goat anti-mouse IgG (H + L) Alexa Fluor 568, and goat anti-rat Alexa Fluor 555 (each at 1:500) (Invitrogen A21434), for 2 h. In parallel, actin was stained with phalloidin iFluor 647 (1:500, ab176759, Abcam, Cambridge, United Kingdom) for 2 h followed by nuclear staining with 20 µM Hoechst 33342 (Thermo Scientific, Waltham, MA, United States) for 5 min. After washing with PBS, samples were mounted with fluorescent mounting medium (Agilent, Santa Clara, CA, United States) and imaged with a confocal laser scanning microscope (LSM 800, Zeiss, Wetzlar, Germany). To quantify protein expression by immunofluorescence, the images of cells stained for the respective protein were evaluated by Fiji (ImageJ 1.53t). For evaluation, each picture was duplicated. One of them was binarized with a very low threshold to define the cell areas. Using the ROI manager of Fiji, the pixels covering the outlined cell areas in the duplicated image were scrutinized for their fluorescence intensities and averaged over the cell area. These values were binned according to their fluorescence intensity/cell area and their frequencies depicted in a histogram. To determine multinuclearity, images of differentiated ER-Hoxb8 cells, the nuclei of which had been stained with Hoechst dye, were counted per OC. Frequency distributions of histograms were statistically compared using the Pearson’s chi-squared test.

### 2.8 Preparation of hydroxyapatite-coated coverslips and resorption assay

13 mm-sized glass coverslips (Fisher Scientific, Schwerte, Germany) were placed in 24-well plates and activated by plasma treatment (100% air, 0.5 mbar) for 2 min in a plasma cleaner (PlasmaFlecto 10, Plasma technology, Herrenberg-Gültstein, Germany). Coverslips were coated with hydroxyapatite as described (Maria et al., 2014) using the following sterile-filtered stock solutions: 8× calcium solution (5.48 M NaCl, 60 mM MgCl_2_, 100 mM CaCl_2_, 50 mM HEPES, pH7.4), 8× phosphate solution (168 mM NaHCO_3_, 44.4 mM Na_2_HPO_4_, pH7.4), and calcium phosphate solution (140 mM NaCl, 4 mM CaCl_2_, 2.25 mM Na_2_HPO_4_, 50 mM HEPES, pH7.4). For 3 days, 500 µL of each freshly 1:8 diluted calcium solution and phosphate solution were added at RT to each well and renewed daily. On day 4, this mixture was replaced by 250 µL calcium phosphate solution for another day. The hydroxyapatite-coated coverslips were washed three times with sterile distilled water, dried at 50°C and sterilized by UV irradiation for 30 min.

To measure osteoclastic resorption, ER-Hoxb8 cells were differentiated on hydroxyapatite-coated coverslips for 10 days. Cells were removed by incubating with 1M NaCl and 0.2% Triton X-100 for 5 min at RT followed by washing four times with distilled water. Areas of resorption were visualized by silver nitrate staining with 200 µL 5% AgNO_3_ per well for 30 min in the dark. After washing once with distilled water for 5 min, addition of 200 µL 5% Na_2_CO_3_ in 4% PFA and UV irradiation for 4 min led to the deposition of silver on hydroxyapatite crystals turning them from dark brown to black. To remove excess undeposited silver, samples were washed three times with distilled water. Samples were directly examined by bright field microscopy or stored under water at 4°C for later examination.

### 2.9 Statistical analysis

All data are mean ± S.E.M. For statistical evaluation, Student’s t-test was carried out using Microsoft Excel. Pearsons Chi^2^-test was used to compare frequency distributions. Differences were considered to be significant with *p* < 0.05, *p* < 0.01, and *p* < 0.0005, and marked with *, **, and ***, respectively.

## 3 Results

### 3.1 *In vitro* differentiation of ER-Hoxb8

**FIGURE 1:**
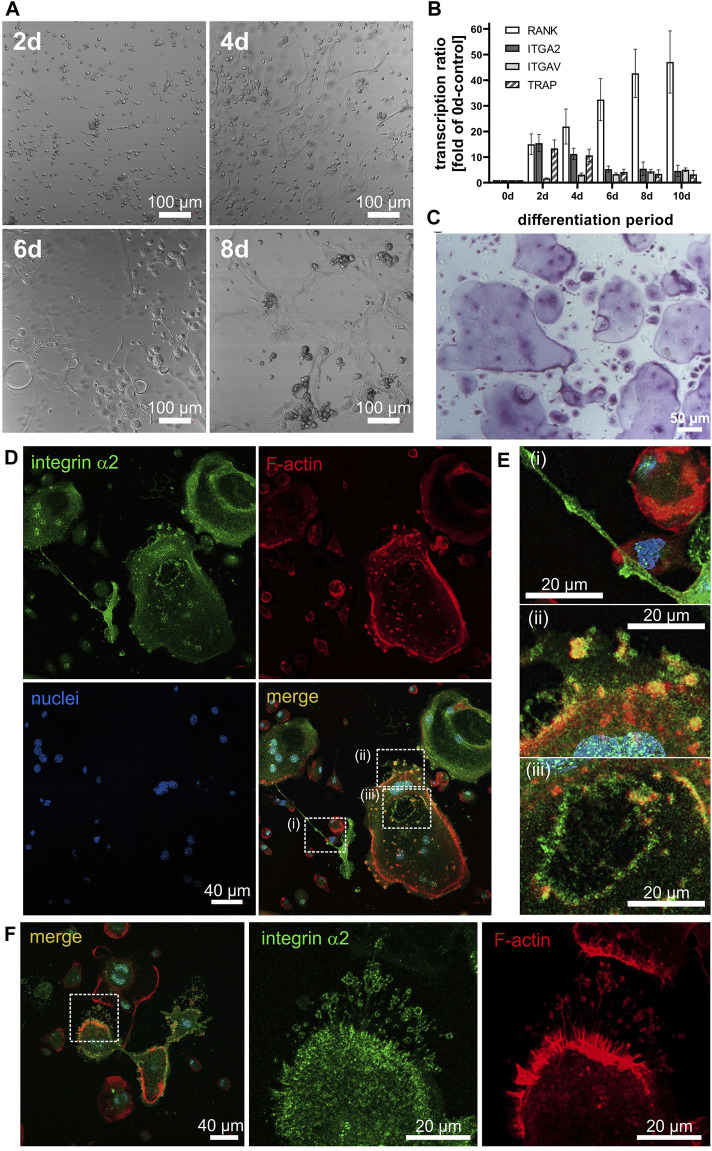
Differentiation of ER-Hoxb8 cells into osteoclasts is accompanied by morphologic changes, increased transcription of marker genes, lacuna formation, and distinct localization of integrin α2β1. **(A)** ER-Hoxb8 cells seeded onto glass coverslips differentiated in response to M-CSF and RANKL. Differential interference contrast images taken with a laser scanning microscope at 10× magnification on the indicated days of differentiation (at least 3 independent experiments). **(B)** At different time points, mRNA was isolated from differentiating ER-Hoxb8 cells, and transcripts of ITGA2, ITGAV, RANK and TRAP were quantified by qRT-PCR (data are mean ± S.E.M. from 10 independent experiments. **(C)** Histochemical staining of TRAP in the resorption lacuna and transcytotic vesicles of ER-Hoxb8-derived osteoclasts on day 7 of differentiation on glass (at least 3 independent experiments). **(D)** Confocal images of ER-Hoxb8 cells differentiated on glass coverslips for 7 days and stained for integrin α2 subunit (green), F-actin (red) and nuclei (blue). **(E)** Enlarged details of regions of interests indicated in **(D)**: Integrin α2β1 in i) tunneling nanotubes, ii) podosomes near the cell periphery, and iii) at the sealing zone of a resorption lacuna. **(F)** Integrin α2 in filopodia. Representative images of n = 4 independent experiments are shown.

Our model cells ER-Hoxb8 proliferate and retain their non-differentiated phenotype in the presence of estradiol. Removal of estradiol and addition of M-CSF and RANKL induced the ER-Hoxb8 cells to differentiate into OCs within 8 days (Figure 1A). To achieve this, they migrated and formed long protrusions as seen on day 4 (Figure 1A). Eventually, they fused into multicellular syncytia (Figure 1A). Osteoclast differentiation on uncoated plastic culture plates was also monitored by transcription analysis of specific markers (Figure 1B). RANK started to be upregulated on the second day and progressively increased thereafter, proving that our ER-Hoxb8 cell culture system recapitulated osteoclastogenesis in the presence of M-CSF and RANKL. The expression of the integrin α2 subunit-encoding ITGA2 gene was already increased by about 15-fold on the second day of differentiation, but decreased again after day 4 (Figure 1B). In contrast, the gene ITGAV encoding the other major integrin α subunit on OCs, αV, was upregulated only 5-fold by day 10 (Figure 1B). Conspicuously, the expression of the OC marker TRAP showed a time course, which peaked around day 2, similar to the one of ITGA2 (Figure 1B). At the protein level, TRAP was detected by histologic staining in the lacuna and in intracellular transcytotic vesicles (Figure 1C). Thus, it could also be detected around the cell nuclei.

### 3.2 Integrin α2β1 is localized in the cell periphery and in protrusions of ER-Hoxb8–derived OCs

To adhere tightly to collagen-I, OCs likely employed integrin α2β1, as this integrin is an high affinity collagen receptor (Madamanchi et al., 2014). In immunofluorescence micrographs, the α2 subunit of integrin α2β1 was localized in the cell perimeter along with the cortical actin network (Figure 1D). In addition, it was present all over the surface along extended cellular protrusions termed tunneling nanotubes, with which OC precursors fused to form syncytia (Figure 1Ei), especially at thicker parts of the nanotubes, where integrin α2β1 is enriched and likely forms precursor adhesion sites. Moreover, podosomes at the cell periphery and underneath the cell soma also contained abundant integrin α2β1 and actin (Figure 1Eii). In particular, a distinct ring of integrin α2β1 characterized the podosome belt surrounding the developing lacunae (Figure 1Eiii). In addition, integrin α2β1 was also present in filopodia, especially at their tips (Figure 1F). The filopodia become especially evident in images of presumably moving osteoclasts, such as in Figure 1F, where the two parts of multicompartmented osteoclasts, connected via short tunneling tube, have actin staining on opposing faces of the syncytium and likely are moving away from each other.

### 3.3 Integrin α2 knockout does not abolish OC differentiation

Based on this subcellular localization and its early upregulation (Figure 1B), we hypothesized that integrin α2β1 is critical for OC differentiation by affecting syncytia formation and bone matrix degradation. To challenge its role in osteoclastogenesis and to unravel the molecular mechanism of its action, we genetically abolished the ITGA2 gene in ER-Hoxb8 cells by using lentiviral CRISPR/Cas9 with a guide RNA homologous to the third exon of mouse ITGA2. Generation of indels was confirmed by T7-endonuclease test (Supplementary Figure S1A). This also prevented the synthesis of an ITGA2-exon3 amplicon with a PCR forward primer corresponding to the guide RNA sequence (Supplementary Figure S1B). Generation of a stop codon in exon 3 was confirmed by sequencing after isolation of genomic DNA from individual cell clones, PCR amplification and TOPO cloning (Supplementary Figure S1C). Accordingly, immunohistochemistry confirmed the absence of integrin α2β1 at the protein level in these ITGA2-KO cells, which showed no signal above background, whereas integrin α2 subunit was abundant on wild type ER-HoxB8 cells (Supplementary Figure S1D). To avoid a possible bias due to the variability between individual cell clones and to ensure intercellular heterogeneity, which is essential for osteoclastogenic cell fusion (Soe, 2020), an ITGA2-KO ER-Hoxb8 population was used instead of individual cell clones. In these cell populations, both non-differentiated ER-Hoxb8 cells and differentiating ITGA2-KO cells showed no detectable amounts of integrin α2 in immunoblots of cell lysates, in contrast to differentiating ITGA2wt cells (Supplementary Figure S1E).

Remarkably, both the wild type population and the ITGA2-KO population of ER-Hoxb8 showed only low fusion rates, presumably due to a low expression of M-CSF receptor (CD115) (Jacome-Galarza et al., 2013). Therefore, we enriched subpopulations of both ITGA2-expressing and ITGA2-deficient ER-HoxB8 cells that abundantly express M-CSF receptor by magnetic-activated cell sorting (MACS). Flow cytometric quantification confirmed the abundance of the M-CSF receptor on the sorted cells. Thus, we obtained a population of ITGA2-KO ER-Hoxb8 cells that expressed the M-CSF receptor at slightly higher level than the ER-Hoxb8 wild type cells (Supplementary Figure S3). Since CD115^high^ cells differentiated with higher consistency after addition of M-CSF, these were used in the following experiments.

OCs have a limited lifespan. To test whether the loss of integrin α2 causes a premature apoptosis during the terminal differentiation of ER-Hoxb8, we carried out a TUNEL assay on ITGA2wt and ITGA2-KO ER-Hoxb8 cells at day2 of differentiation. However, there was no significant difference in the percentage of apoptotic cells between the 2 cell types, suggesting that integrin α2β1does not affect the survival of OCs (Supplementary Figure S2).

We also tested whether ITGA2-KO cells express more integrin αVβ3 in a compensatory manner. As detected by immunofluorescence, after 6 days of differentiation, ITGA2-KO cells expressed slightly more integrin αVβ3 than the ITGA2wt cells (Supplementary Figure S2B–D). However, the expression of this RGD-dependent integrin slowly increased, after the expression of collagen-binding integrin α2β1 had already peaked at day 2 (Figure 1B).

### 3.4 OSCAR does not compensate for the missing collagen receptor in ER-Hoxb8 ITGA2-KO cells

**FIGURE 2:**
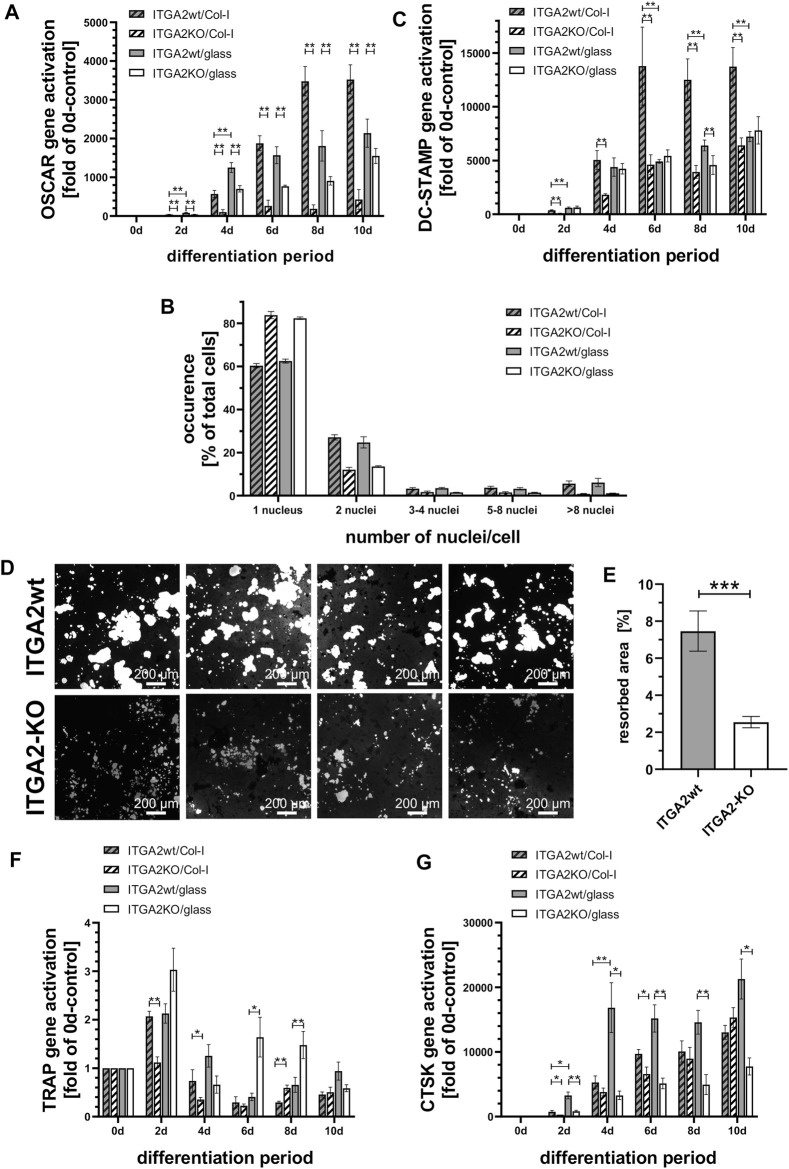
Integrin α2β1 deficiency cannot be compensated by OSCAR and results in reduced osteoclastic syncytia formation and hydroxyapatite resorption. **(A)** qRT-PCR of OSCAR at different time points of differentiation of ER-Hoxb8 ITGA2wt and -ITGA2-KO cells on collagen-I-coated or uncoated glass coverslips (n = 6 and 8, respectively). **(B)** Quantification of cell fusion after 7 days of differentiation via the number of Hoechst 33342-stained nuclei per cell. More than 7,500 cells in 3 independent experiments were analyzed. A Pearson Chi-squared test revealed a significant difference between the ITGA2wt and ITGA2-KO cell populations (*p* < 0.0001), but no significant difference for each of the 2 cell variants between collagen-I and uncoated glass. **(C)** qRT-PCR of DC-STAMP at different time points of differentiation of Hoxb8-ITGA2wt and -ITGA2-KO cells on collagen-I-coated or uncoated glass coverslips. **(D)** Resorption areas (light grey) of ER-Hoxb8-derived osteoclasts after 10 days of differentiation on hydroxyapatite-coated coverslips. Cells were removed and remaining hydroxyapatite was silver-stained. Representative images of 8 independent experiments are shown. **(E)** Biometric quantification of resorption areas in **(D)** using the trainable WEKA segmentation of Fiji (ImageJ 1.53t), n = 8 ± S.E.M., *p* < 0.05. **(F)** qRT-PCR of TRAP at different time points of differentiation of ITGA2wt and ITGA2-KO ER-Hoxb8 cells on collagen-I-coated or uncoated glass coverslips. **(G)** qRT-PCR of CTSK at different time points of differentiation of ITGA2wt and -ITGA2-KO cells on collagen-I-coated or uncoated glass coverslips. qRT-PCR data (panels **C**, **F**, and **G**) are represented as mean ± S.E.M. from three to four independent experiments in duplicate with Student’s t-test showing pairwise significances as *p* < 0.05 (*) and *p* < 0.01 (**).

Genetic ablation of the ITGA2 gene may cause compensatory upregulation of other collagen receptors. None, of the other three collagen-binding integrins were likely candidates for compensation, as we could not detect integrin α1 on these cells. Nor are the other two integrins that bind to triple-helical collagen directly and with high affinity, α10β1 and α11β1, characteristically expressed in chondroyctes and (activated) fibroblasts, respectively, but not on monocytic cells (Heino, 2014; Lundgren-Akerlund and Aszodi, 2014; Zeltz et al., 2022). Hence, OSCAR appeared to be a more likely candidate for a compensatory mechanism. To check whether OSCAR, as alternative collagen receptor, would compensate for the loss of integrin α2β1, we analyzed its expression. However, differentiating ER-Hoxb8 ITGA2-KO cells upregulated OSCAR transcription significantly less than the integrin α2β1-expressing ER-Hoxb8 cells, particularly when grown on collagen-I-coated glass coverslips (Figure 2A). Remarkably, ligand occupancy of integrin α2β1 promoted OSCAR gene activation after 4 days, with OSCAR gene expression of ITGA2wt cells being always higher on collagen-I than on uncoated glass. Compared to ITGA2 activation, OSCAR gene expression increased noticeably later, only after 4 days, and peaked on day 8 (Figure 2A). The reduced gene activation in ITGA2-KO cells and its late onset ruled out that OSCAR can compensate for integrin α2β1 deficiency early in osteoclastogenesis.

### 3.5 Integrin α2β1 deficient OC precursor cells show less syncytia formation and hydroxyapatite resorption

Loss of integrin α2 impaired the formation of syncytia. We quantified this effect on the fourth day of OC differentiation by counting the multinucleated cells and the number of their nuclei in microscopic images. Irrespective of whether the glass coverslips were or were not coated with collagen-I, significantly less ER-Hoxb8 ITGA2-KO formed syncytia (17%, while 83% remained mononuclear) than integrin α2β1-expressing ER-Hoxb8 cells, which formed syncytia more than twice as often (39%; while 61% remained mononuclear). The proportion of multinucleated cells with 2, 3-4, five to eight or more nuclei was always higher in the integrin α2β1-expressing cells (Figure 2B).

Mechanistically, syncytia formation requires an enhanced expression of DC-STAMP, a transmembrane protein that contributes to cell fusion. It was highly upregulated in ER-Hoxb8 ITGA2wt cells during days 4–10, particularly on collagen-I but not on uncoated glass (Figure 2C). In contrast, ITGA2-KO cells upregulated the transcription of DC-STAMP during osteoclast differentiation significantly less and DC-STAMP expression remained almost unaffected by the presence of the collagen substrate (Figure 2C). Thus, integrin α2β1 promoted collagen-I dependent expression of DC-STAMP.

To test the functional ability of the OCs to resorb bone matrix, ER-Hoxb8 cells were differentiated for 10 days on crystals of the bone mineral hydroxyapatite previously grown on coverslips. Subsequently, non-resorbed hydroxyapatite was detected after silver staining with a bright field microscope (Figure 2D). Quantification of the resorption areas revealed that the ITGA2-KO cells resorbed far less hydroxyapatite than the integrin α2β1-expressing cells (Figure 2E).

In line with this, the expression of TRAP, which is involved in hydroxyapatite dissolution (Hayman, 2008), was significantly higher in ITGA2wt cells on collagen-I than on glass for the first 4 days (Figure 2F). Conversely, expression levels of TRAP in ITGA2-KO cells were higher on glass than on collagen-I, particularly on days 6 and 8. The latter could be caused by vitronectin of the culture medium, which stimulates TRAP expression in an integrin αVβ3-dependent manner (Chambers and Fuller, 2011).

Cathepsin K that also contributes to bone matrix degradation (Dai et al., 2020) was similarly expressed by ITGA2wt and ITGA2-KO on collagen-I (Figure 2G). However, ITGA2wt cells activated the CTSK gene encoding cathepsin K significantly more on uncoated glass than on collagen-I, suggesting that integrin α2β1 can downregulate cathepsin K transcription ligand-dependently (Figure 2G). Taken together, these data show, that integrin α2β1 expressed early in osteoclastogenesis regulates the expression of bone matrix degrading enzymes, such as TRAP and cathepsin K, in a partially ligand-dependent manner, thereby promoting the resorption of hydroxyapatite.

### 3.6 Integrin α2-KO cells spread less, but with more and spikier protrusions than ITGA2wt cells

**FIGURE 3:**
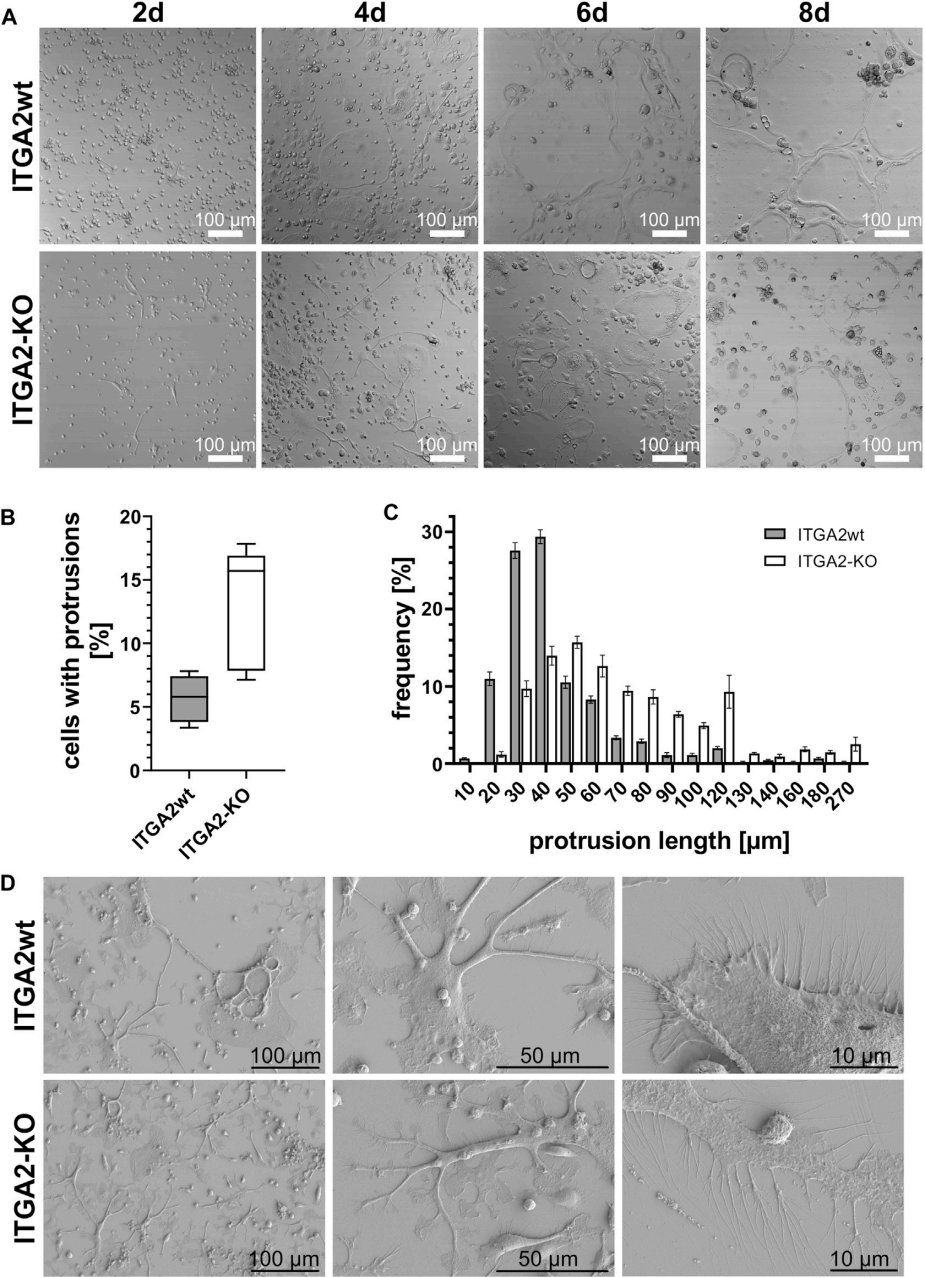
During OC differentiation, ITGA2-expressing and ITGA2-deficient OC precursor cells differ in the number and length of their protrusions. **(A)** Differential interference contrast images of ITGA2wt and ITGA2-KO ER-Hoxb8 cells on collagen-I-coated glass coverslips were taken with a laser scanning microscope at 10× magnification. Representative images of at least 3 independent experiments are shown. **(B)** Number and **(C)** lengths of cellular protrusions of ITGA2wt and ITGA2-KO cells at day 4 of differentiation were quantified with the Simple Neurite Tracer (SNT) module of Fiji (ImageJ 1.53t). Data are mean ± S.E.M. from 5 independent experiments. Box-plot of protrusion numbers with 25- and 75 percentiles is shown in **(B)** and differed significantly between the 2 cell variants when compared by Student’s t-test (*p* = 0.0129). Protrusion lengths were binned according to the indicated classes. Frequency distributions between ITGA2wt and ITGA2-KO cells were significantly different according to the Pearson Chi^2^ test (*p* < 0.0001, n = 1,198 cells) **(D)** Representative scanning electronic images of ITGA2 expressing and deficient osteoclastic cells at day 4 of differentiation on collagen I-coated wafers.

In order to better understand the processes leading to cell fusion, we also examined the involvement of integrin α2β1 in protrusion formation that precedes cell fusion. For this purpose, ITGA2-KO *versus* ITGA2wt ER-Hoxb8 cells were monitored during osteoclastic differentiation by real time video microscopy (Suppl. Videos: ITGA2wt and ITGA2 knockout). Irrespective of whether or not the coverslip had been coated with collagen-I, both integrin α2β1-expressing and–deficient cells underwent OC differentiation, although ITGA2-KO cells differentiated slower than ITGA2wt cells with respect to morphologic changes (Figure 3A). Both cell variants formed extended cell protrusions, resembling tunneling nanotubes (TNT) (Dupont et al., 2018), albeit with striking differences. Integrin α2β1-deficient cells did not fully spread and formed more protrusions that were longer than those of integrin α2β1-expressing cells (Figures 3B,C). Quantification revealed that only 5.6% ± 1.8% of the wild type cells formed protrusions, but 13.0% ± 4.8% of ITGA2-KO cells. The protrusions of the latter had a median length of 57.3 µm and differed significantly from the shorter protrusions of the ITGA2wt cells with a median length of 33.2 µm (Figure 3C).

These protrusions were studied at high resolution by scanning electron microscopy on day 4 of differentiation on collagen-I-coated silicon wafers (Figure 3D). Integrin α2β1-deficient osteoclastic cells spread less than ITGA2 expressing cells. Strikingly, the long and spiky TNTs of ITGA2-KO cells showed flattened paddle-like planar extensions. They were interrupted by concave areas that gave the protrusions the appearance of twigs with single leaves. At higher resolution, the numerous filopodia of the osteoclastic cells showed significant integrin α2β1-dependent differences. While filopodia of ITGA2wt cells were hardly branched and pointed straight away from the cell, the corresponding protrusions of ITGA2-KO cells were longer and more often dichotomous or even more branched, giving them a twisted appearance (Figure 3D, right panels).

### 3.7 ER-HoxB8 ITGA2-KO cells do not upregulate tetraspanin CD9 during differentiation

**FIGURE 4:**
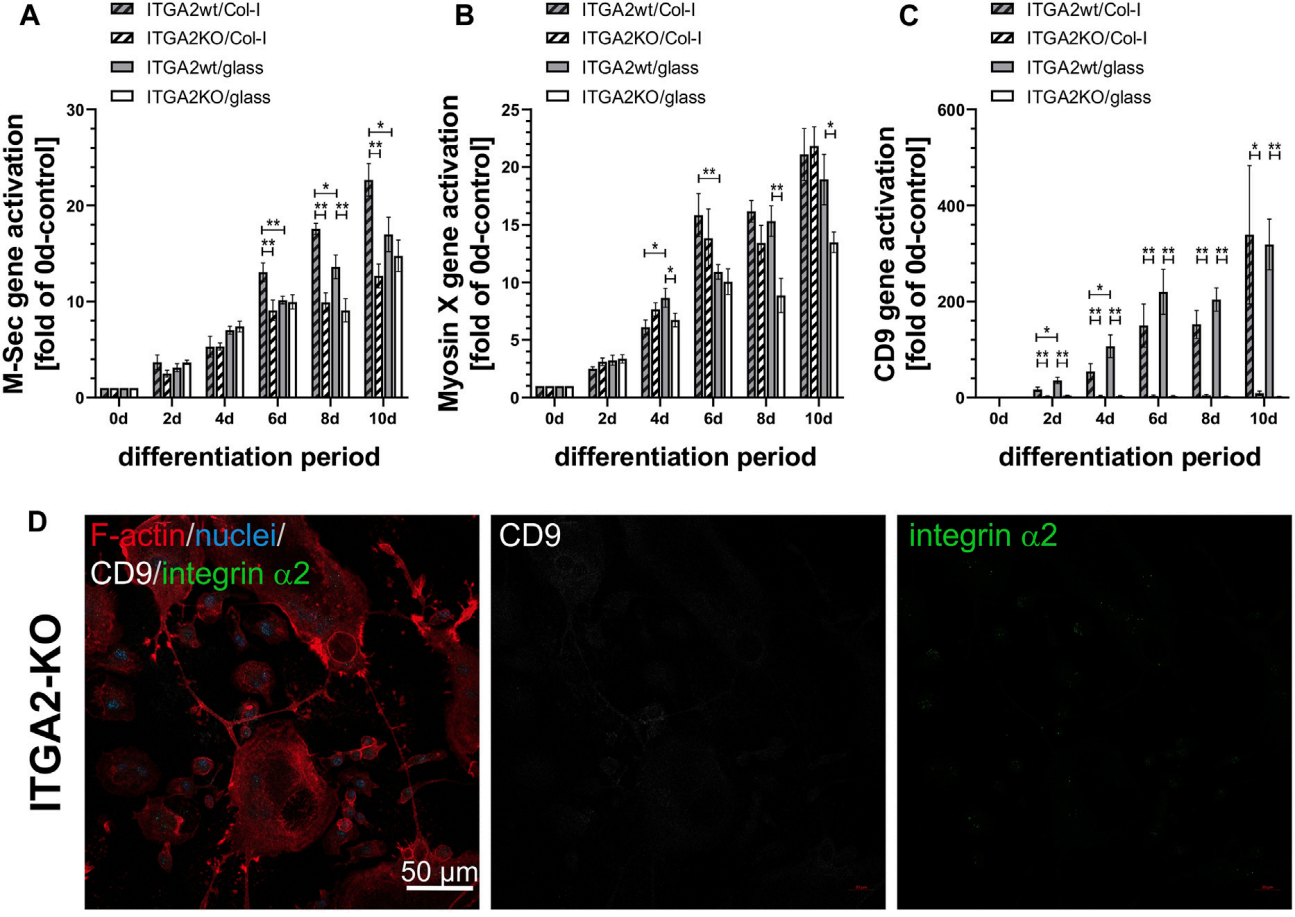
Expression of osteoclastic marker genes involved in formation of cellular protrusions and syncytia formation. qRT-PCR data of **(A)** M-Sec that plays a role in TNT formation and fusion, **(B)** myosin X that is localized on the tips of filopodia, in lamellipodia and podosomes, and **(C)** the tetraspanin CD9 that contributes to filopodia formation and cell fusion during osteoclastogenesis. Data are mean ± S.E.M. from 3 independent experiments in duplicate with Student’s t-test showing pairwise significances as *p* < 0.05 (*) and *p* < 0.01 (**). **(D)** CD9 deficiency in ITGA2-KO osteoclasts at day 6 of differentiation on collagen-I shown by confocal laser scanning microscopy at 20× magnification. F-actin (red), integrin α2 (green), CD9 (grey), nuclei (blue). Representative images from one out of 4 experiments are shown.

To study the cellular protrusions mechanistically, we analyzed the expression of protrusion-related proteins, such as M-Sec, myosin X and the tetraspanin CD9 during osteoclast differentiation (Figures 4A–C). We used M-Sec as a marker of the formation of TNTs (Takahashi et al., 2013), while myosin X is a motor protein found in both TNTs and filopodia (Tasca et al., 2017). However, quantification of gene expression by qRT-PCR showed a significant impairment of upregulation of M-Sec in the absence of integrin α2 or collagen-I only at days 6–10 of differentiation, whereas ITGA2-KO cells had already formed more cellular protrusions than ITGA2wt cells during the early days of differentiation (Figure 3A). Myosin X was transcriptionally upregulated in both ITGA2wt and ITGA2-KO cells cultured on collagen-I, albeit with slightly lower myosin-X expression in ITGA2 KO cells. This difference became more apparent on uncoated glass at later stages of differentiation (Figure 4B).

In strong contrast to M-Sec and myosin X, the expression of tetraspanin CD9 essentially required the presence of integrin α2β1, as CD9 was not upregulated at all in integrin α2β1-deficient cells during differentiation, whereas the transcription of the CD9 gene progressively increased more than 300-fold in ITGA2wt cells (Figure 4C). CD9 transcription was upregulated exclusively in ITGA2wt, irrespective of collagen-I or glass substratum, suggesting that the activation of the CD9 gene depended solely on the presence of integrin α2β1, but not on its binding to collagen-I. No CD9 protein was detectable on ITGA2-KO cells by immunofluorescence microscopy (Figure 4D).

### 3.8 CD9 shows a distinct cellular localization that only partially overlaps with the one of integrin α2β1

**FIGURE 5:**
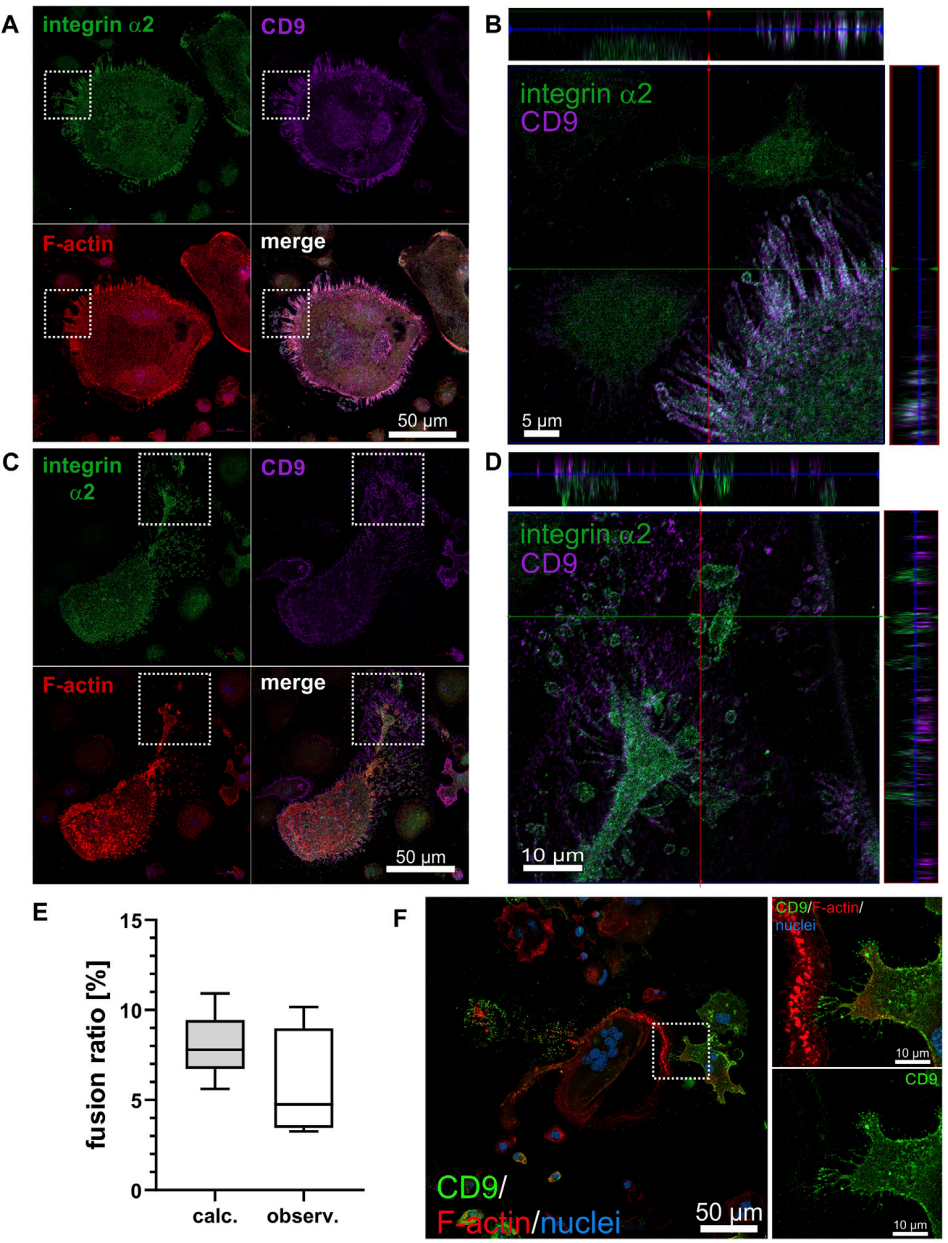
CD9 only partially colocalizes with integrin α2β1 and its heterogeneous expression on OC precursor determines intercellular contact formation and cell fusion in osteoclastogenesis. **(A)** Representative image of an ITGA2 expressing non-polarized, rounded OC at day 6 of differentiation on collagen-I showing marked colocalization of integrin α2, CD9 and F-actin in protrusions. **(B)** Orthogonal view of cropped region marked in **(A)** with three-fold stretched *z*-axis to better discern the colocalization of integrin α2 and CD9. **(C)** Reduced co-localization of integrin α2 and CD9 in filopodia of a polarized (likely motile) OC. **(D)** Orthogonal view of cropped region marked in **(C)** with three-fold stretched *z*-axis to better discern the localization of integrin α2 and CD9 in different planes, with integrin α2 closer to the substrate and CD9 more distal from the substrate. Representative confocal laser scanning microscope image acquired at 40× magnification with integrin α2 (green), CD9 (purple), F-actin (red), nuclei (blue). **(E)** The ratio of bicellular fusion of heterogeneously CD9 expressing cells to homogeneously CD9-positive cell pairs was determined in two ways. Theoretically, the fusion ratio was calculated using the determined frequency of mononuclear CD9-expressing ITGA2wt cells (4,235 cell from 66 images, bin size: at least 30 cells) as a fully stochastic two-step Bernoulli process. Experimentally, binuclear cell fusion events from ITGA2wt cells grown on collagen-I for 4 days were quantified after immunostaining for CD9 expression and the fusion ratio of heterogeneously CD9-expressing cells to homogenously CD9-positive cells was calculated (>300 binuclear cell fusions on 66 images, with a bin size of at least 9 binuclear syncytia). With a significance level of *p* = 0.05, the experimentally determined binuclear fusion ratio did not differ significantly from the theoretically calculated one, suggesting that binuclear fusion of differentiating ER-Hoxb8 cells is a stochastic process, independently of cellular CD9 abundance. **(F)** Intercellular contact sites of fusing ER-Hoxb8 cells may differ with respect to the presence of CD9. After 4 days of differentiation on collagen-I, OCs were immunofluorescently stained for CD9 (green), F-actin (red), nuclei (blue) (left panels). The enlarged section on the right shows a subcellular contact site of two fusing cells (top), which are heterotypic with regard to CD9 expression (bottom, single channel). A representative confocal laser scanning microscope image acquired at 20× magnification from n = 4 independent experiments from a total of more than 40 arbitrarily selected contacting cells is shown.

Activation of the CD9 gene was linked to the presence of integrin α2β1. As CD9 can associate with integrin α2β1 at the protein level (Reyes et al., 2018), we analyzed the distribution for both proteins, CD9 and integrin α2β1, in differentiated ER-Hoxb8 ITGA2wt cells by immunofluorescence (Figures 5A–D). The abundance of CD9 within the cell population was heterogeneous, inasmuch as some cells expressed more and other ones almost no CD9. In roundish cells with no leading edge, CD9 colocalized with integrin α2β1 in filopodia along with actin (Figures 5A,B). In contrast, in cells with a polarized shape, the colocalization of CD9 with integrin α2β1 was strongly reduced (Figure 5C). Both proteins were found in TNTs and filopodia, but not in close proximity as they were detected in different z-stacks of confocal images, ruling out a direct physical association of CD9 and integrin α2β1 at these sites (Figure 5D).

The heterogeneous expression of CD9 in ER-Hoxb8 ITGA2wt cells prompted the question whether different abundance of CD9 on cells may favor the fusion between cells. To test this hypothesis, we measured the relative frequency of CD9-expressing cells in the population of differentiation ITGA2wt cells on collagen-I on day 4 by immunofluorescence staining for CD9. Among more than 4,000 cells on more than 60 microscopic images, we calculated the relative frequency of CD9-positive cells to be 20.7% ± 3.7%. Assuming that the fusion of the OC precursor into a binuclear syncytium is a Bernoulli process of two independent, memoryless steps with a binary outcome (CD9-positive or CD9-negative), we determined the ratio of heterogenous cell fusion (with one of 2 cells is CD9-positive) to the homogenous cells fusions of two CD9-positive cells both theoretically and experimentally (Figure 5E). The latter was quantified by immunofluorescent staining for CD9 of ER-Hoxb8 ITGA2wt cells having differentiated on collagen-I for 4 days. Among the binuclear cells, the ratio of CD9-heterotypic cell fusions to CD9-positive homotypic fusions was 5.73% ± 3.09%, which is not significantly different from the theoretical value of 7.94% ± 1.71%, calculated for a stochastic Bernoulli process (Figure 5E). Hence, the binuclear fusion of ER-Hoxb8 cells is a stochastic process with respect to CD9 abundance, which does not determine the preference of a differentiating ER-Hoxb8 ITGA2wt cell for a CD9-positive or CD9-negative fusion partner. Nonetheless, it is noteworthy, that the subcellular sites of contacts between fusing cells often showed a staining contrast for CD9, as protrusions with high abundance of CD9 frequently approached CD9-poorer sites of partner cells (Figure 5F).

### 3.9 The osteoclastogenesis-promoting effect of integrin α2β1 is not mediated by changes in RANK and NFATc1 expression

**FIGURE 6:**
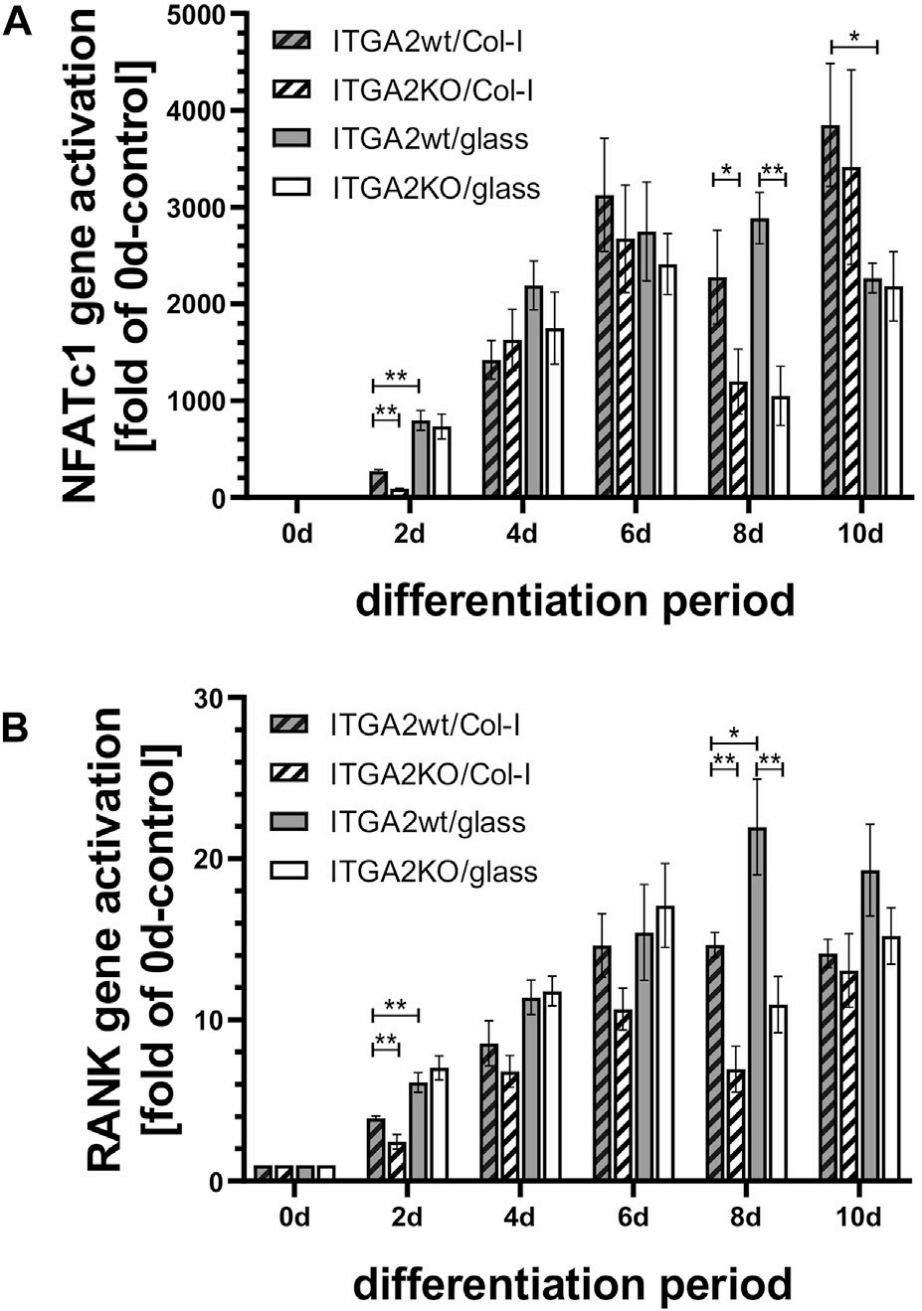
Expression of the differentiation markers NFATc1 and RANK during OC differentiation is similar in ITGA2wt and ITGA2-KO cells. qRT-PCR data of **(A)** NFATc1 and **(B)** RANK in ITGA2wt and ITGA2-KO cells during differentiation on collagen-I coated or uncoated glass coverslips. Data are mean ± S.E.M. from three to four independent experiments in duplicate, with Student’s t-test showing pairwise significances as *p* < 0.05 (*) and *p* < 0.01 (**).

Presence of integrin α2β1 increased gene activation of CD9, DC-STAMP, TRAP and OSCAR, whereas CTSK gene transcription was reduced. The expression of DC-STAMP, OSCAR and CTSK depended on the collagen-I substratum, whereas the activation of the TRAP and CD9 genes were independent of the integrin α2β1 ligand. To get insights into the underlying signaling mechanism of α2β1 integrin-regulated gene activation, we tested whether the major osteoclastogenic signaling pathways involving RANK and NFATc1 were influenced by the presence or absence of the integrin α2 subunit (Figure 6). The master regulator for osteoclastogenesis, NFATc1, was strongly expressed from day 2 on and reached an expression peak of about 3000-fold on day 6 in both ITGA2wt and ITGA2-KO cells on collagen-I. However, its upregulation did not consistently depend on glass or collagen-I substrate along the course of differentiation. RANK, an essential signaling receptor for osteoclastogenesis, showed transient differences in its expression at days 2 and 8 similar to NFATc1. However, neither NFATc1 nor RANK were expressed with consistently major differences in the ITGA2-KO *versus* ITGA2wt cells during the entire course of differentiation. This was in line with our finding that loss of integrin α2β1 did not completely abolish osteoclast differentiation in general, but rather impaired key substeps of differentiation. Based on several complementary observations, such as morphologic changes, activation of osteoclastic marker genes, and characteristic cell functions (formation of TNTs, cell fusion, resorption of hydroxyapatite), we found that differentiation to osteoclasts also occurs in ITGA2-KO cells. However, the resulting ITGA2-KO osteoclasts showed striking morphological changes compared with ITGA2wt osteoclasts and were impaired in the cell fusion and hydroxyapatite resorption. In particular, the comparative observation of OC differentiation on collagen I-coated *versus* uncoated substrates suggests that cell spreading, protrusion formation, cell fusion and resorptive functions are regulated by integrin α2β1 and its interaction with collagen-I.

## 4 Discussion

The exact nature of the involvement of integrin α2β1 in osteoclastogenesis is not yet fully understood. Work on OCs is hampered by the fact that they cannot be easily isolated from bone. Harvesting of differentiating or differentiated primary cells and their subsequent cell biological/biochemical characterization *ex vivo* is practically impossible due to (i) the low number of primary monocytes in the blood and bone marrow, (ii) the low frequency of monocyte fusion to OCs and (iii) the fragility of the resulting multinuclear giant osteoclast cells. Also, *in vitro* models for OC differentiation, including models, in which osteoclastic precursor cells can be genetically modified, are rare. For this study, we employed ER-Hoxb8 cells, which are conditionally immortalized murine monocytic precursor cells that, due to their estrogen-dependent expression of transcription factor Hoxb8, differentiate into OCs *in vitro* only upon removal of estradiol and addition of the cytokines, M-CSF and RANKL (Wang et al., 2006; Di Ceglie et al., 2017). The differentiation rate into OCs in our model were comparable to the ones of primary monocytic cells from mouse or murine blood (Stange et al., 2013; Chandrabalan et al., 2024). Using lentiCRISPR/Cas9-technology, we knocked out integrin α2 in ER-Hoxb8 cells (Sanjana et al., 2014; Di Ceglie et al., 2017), while keeping them in a non-differentiating state of proliferation. When comparing these ER-Hoxb8 ITGA2-KO cells with integrin α2-expressing ER-Hoxb8 cells under differentiating conditions, we found that the integrin α2 deficient cells underwent osteoclast differentiation, albeit not to full extent and function. They showed several differences in cell morphology, expression of osteoclastic genes, cell fusion, and lacuna function.

Integrin α2β1 is a collagen-I receptor with a broad tissue distribution and found in epithelial cells, fibroblasts, platelets (Adorno-Cruz and Liu, 2019) and in particular on OCs, in which integrin α2β1 binds to collagen together with the OC-specific collagen receptor OSCAR (Nedeva et al., 2021). In our study, we provide evidence that the gene ITGA2 encoding the integrin α2 subunit, is activated in OC precursor cells at the onset of differentiation. In contrast, transcriptional activation of the OSCAR gene occurs later and is promoted by the previously expressed integrin α2β1 and its binding to collagen-I. After collagen binding, OSCAR associates with the FcγR and triggers an intracellular increase in Ca^2+^, thereby activating the NFATc1 gene (Nedeva et al., 2021). In line with this, we show that the expression pattern of OSCAR resembles the one of NFATc1 at differentiation days 8–10. The onset of osteoclastogenesis before the increase of OSCAR expression and the early sharp increase in ITGA2 expression as early as day two suggest that integrin α2β1 is a crucial receptor for collagen-induced differentiation steps. Among other proteins, integrin αVβ3 is considered as a marker of mature osteoclasts (Anwar et al., 2023). Under osteoclastogenic conditions, freshly isolated bone marrow macrophages express over a time course of 10 days, like in our study, initially no αVβ3 but α5β3 (Teitelbaum, 2000). With the development of the osteoclast phenotype, the integrin subunit β5 is replaced by β3, and mature osteoclasts express only αVβ3 but no α5β3 (Teitelbaum, 2000). The time course of integrin expression we observed is also consistent with a previously reported 11-day time course of osteoclastogenesis of macrophages from murine bone marrow, where β3 mRNA was detectable by Northern blot from day 5 onwards (Inoue et al., 1998). Due to a higher sensitivity of qRT-PCR, we could detect the onset of integrin αV expression earlier, as early as day two of differentiation. The expression of the integrins, α2β1 and αVβ3, were reported to be linked in a compensatory mechanism (Horton et al., 2003; Chambers and Fuller, 2011). We observed that, during OC differentiation, the expression of integrin αVβ3 increased progressively, but slowly, and without a peak. After knockout of the ITGA2 gene, integrin αVβ3 increased slightly in a compensatory manner on the cells. However, integrins, α2β1 and αVβ3, bind different ECM ligands. Type I collagen is one of the major components of the bone ECM (Florencio-Silva et al., 2015) and the ligand of integrin α2β1, while other bone matrix proteins, such as osteopontin, are recognized by integrin αVβ3 on OC (Nakamura et al., 2007).

In differentiating ER-Hoxb8 cells, integrin α2β1 was localized to belt-like podosome rings around developing lacunae. Besides, integrin α2β1 was detected in several types of cellular protrusions, such as filopodia and TNTs. Both podosomes and filopodia play important roles in substrate sensing and adhesion as well as cell migration, cell fusion and formation of the resorption lacuna’s sealing zone (Song et al., 2014). In particular, the formation of TNTs is essential for osteoclastogenesis as these long bridges are important for cell-cell communication between OC-precursor cells and contribute to cell fusion (Takahashi et al., 2013; Georgess et al., 2014; Dupont et al., 2018; Dufrancais et al., 2021). Therefore, it is astonishing that loss of integrin α2β1 resulted in extended cellular protrusions in osteoclastic ER-Hoxb8 cells. Moreover, despite forming more and longer TNTs with more branched filopodia, integrin α2β1-deficient ER-Hoxb8 cells fused less into syncytia. Scanning electron microscopy visualized an unusual lateral flattening of the TNTs of integrin α2β1-deficient cells into small paddle-like areas, which was associated with an increased protrusion length of ITGA2-KO cells. This may represent a compensatory attempt by the cells to find a non-collagenous substrate that enables them to adhere firmly. Strikingly, integrin α2-deficient cells failed to upregulate the tetraspanin CD9, which reportedly is also involved in formation of cell membrane protrusions, integrin-dependent cell migration, fusion during osteoclastogenesis, and cell signaling (Berditchevski, 2001; Ishii et al., 2006; Iwai et al., 2008; Termini and Gillette, 2017). A recent paper pinpointed CD9 as a driving force to initiate protrusion formation (Manzano et al., 2022). In contrast to this, we observed more and longer protrusions in ITGA2-KO cells along with low CD9 expression. In line with the referred paper (Manzano et al., 2022), we also showed that low expression of CD9 caused decreased cell fusion. Our data also demonstrated that, in differentiating OCs, CD9 does not associate exclusively with integrin α2β1 at the protein level. As CD9 can associate with other membrane proteins to form tetraspanin-enriched microdomains (TEMs) that fulfil pleiotropic functions (Reyes et al., 2018), our results suggested that the composition of these TEMs varied during OC differentiation and did not necessarily include integrin α2β1. In contrast to CD9, M-Sec and myosin X did not show remarkable expressional differences in correlation with integrin α2β1. M-Sec contributes to the formation of TNTs, along which DC-STAMP is transported from the cell soma to the tips of the protrusions (Takahashi et al., 2013). Myosin X triggers formation of filopodia and regulates integrin activity there (Takahashi et al., 2013; Miihkinen et al., 2021). Although we found reduced upregulation of M-Sec in the absence of integrin α2 or collagen-I in the later phase of differentiation, this occurred after protrusions had formed. Myosin X gene activation was only slightly reduced in the absence of integrin α2.

Although the CD9 gene was transcriptionally activated exclusively in integrin α2β1-expressing OC precursors, the integrin α2β1-dependent CD9 gene activation did not depend on collagen-I as cell substrate, maybe due to the fact that CD9 may associate with integrin α2β1 in CD9-containing TEMs that act as signaling hubs (Hemler, 2005; Brosseau et al., 2018; Reyes et al., 2018), independently of ligand occupancy of the integrin. Reportedly, CD9 functionally associates with FcγRs on macrophages (Kaji et al., 2001), similar to OSCAR (Nedeva et al., 2021). Integrin α2β1 can also associate with CD9, although our immunofluorescence data showed only a partial overlap of colocalization of CD9 with the integrin α2 subunit. Instead, the two proteins localized distinctly alongside and on the tips of filopodia and TNTs. Furthermore, both CD9-integrin α2β1 association and rearrangement of CD9-assembled TEMs transiently depended on the differentiation state of the OCs. Therefore, CD9, like other tetraspanins, may contribute differently to the individual steps of osteoclastogenesis, such as monocyte homing to the bone marrow (Leung et al., 2011), integrin-mediated cell migration (Powner et al., 2011) and signaling processes (Yi et al., 2006).

Integrin α2β1-deficient ER-HoxB8 cells fused to syncytia at a lower rate. CD9-containing TEMs either promote or inhibit membrane fusion of different cell types (Brosseau et al., 2018; Reyes et al., 2018). The presence of CD9 on a murine egg cell reportedly supports fusion with a sperm cell, like the homotypic CD9-dependent fusion of myoblasts into myofibers (Tachibana and Hemler, 1999; Zhu et al., 2002). Conversely, CD9-inhibiting antibodies increased fusion of blood monocytes into multinucleated giant cells (Takeda et al., 2003; Parthasarathy et al., 2009). Here we show, using bone marrow-derived ER-Hoxb8 cells, that inhibition of CD9 expression by knockout of ITGA2 significantly impeded the fusion of osteoclast precursor cells into multinucleated giant OCs. This is in line with a report that a CD9-specific antibody significantly inhibits multinucleated OC formation from bone marrow monocytes (Yi et al., 2006). The different repertoire of integrins or cell-specific differences in TEM compositions on the human blood monocytes (Takeda et al., 2003; Parthasarathy et al., 2009) *versus* bone marrow derived cells may account for these distinct effects of CD9 on cell fusion. In addition, as the transcriptional abrogation of CD9 in ITGA2-KO cells did not abolish cell fusion entirely, there must be additional and alternative proteins that contribute to syncytia formation, such as the fusion-supporting DC-STAMP (Yagi et al., 2005). Here, we demonstrated that the DC-STAMP gene is transcriptionally activated strongly by the presence of integrin α2β1 in a collagen-I-dependent manner. Hence, the early expression of integrin α2β1 and α2β1-mediated adhesion of OC precursor cells to collagen-I are prerequisites for the expression of DC-STAMP, which together with the integrin α2β1-dependent CD9 expression promoted osteoclastic cell fusion.

In addition to the observed disturbances in protrusion formation and cell fusion, we observed that ITGA2-KO OCs were compromised in their ability to resorb hydroxyapatite, which may partially be a consequence of reduced osteoclastic cell fusion. To date, there is little known about the signals that trigger the bone resorbing activity of OCs. Both integrins, αVβ3 and α2β1, likely induce bone resorption (Chambers and Fuller, 2011). Here, we show that activation of the resorption-relevant genes of TRAP and cathepsin K depended on integrin α2β1. In ITGA2wt cells, TRAP showed a time course of expression remarkably similar to the ITGA2 gene, implicating that integrin α2β1 promotes TRAP gene expression. The early gene activation of TRAP correlates with its pleiotropic functions, among which are the cytoplasmic production of reactive oxygen species, the processing of collagen and dephosphorylation of osteopontin, which is important for OC migration (Hayman, 2008). Although cathepsin K contributes to bone matrix degradation (Dai et al., 2020), our data showed no gross differences in its expression in ITGA2wt versus ITGA2-KO cells when differentiated on collagen-I. In contrast, this difference was profoundly enhanced when these cells were grown on glass, suggesting that integrin α2β1 may curb cathepsin K gene activation in a collagen-I-dependent manner. Moreover, additional or other factors might promote cathepsin K expression in ITGA2wt cells. This might be CD9-induced signaling events, as the expression pattern of cathepsin K resembles the one of CD9 in ER-Hoxb8 ITGA2wt cells with respect to the time course of differentiation.

Also with respect to cell fusion and lacuna formation, other studies demonstrated that compensatory upregulation of integrin α2β1 in Glanzmann thrombasthenia patients lacking integrin αVβ3 restored the full activity of OCs (Horton et al., 2003). As both integrins support OC differentiation, dysfunction of either of the two integrins or of the signal transduction via integrin-linked kinase manifests in osteopetrosis (Blair et al., 2009; Dossa et al., 2010). Also via integrin α2β1, other cells within the bone contribute to a functional bone turnover, as overexpression of the ITGA2 gene promotes bone marrow stromal cells and osteoblast function (Hu et al., 2013). Former studies on platelets correlated thrombosis risk with the abundance of integrin α2β1 controlled by genetic polymorphism of the ITGA2 gene (Kunicki and Nugent, 2002). The fact that our data show a strong linkage between the presence of integrin α2β1 with CD9 in differentiating OCs, would provide a rationale to extent the polymorphism studies of ITGA2 on platelets to patients with dysfunctional bone turnover and to correlate the different ITGA2 alleles to the risk of osteoporosis or osteopetrosis in aged patients. The clinical relevance is even more evident, as CD9 is abundantly expressed in OCs in osteoporosis and contributes to bone erosions of collagen-induced arthritis (Iwai et al., 2008).

In conclusion, integrin α2β1 regulates the activity of OCs inter alia by activating genes, which are relevant in the differentiation into OCs, such as DC-STAMP and TRAP. Overall, our data show that integrin α2β1 is substantially, although not necessarily, involved in osteoclast formation. The integrin α2β1, through its early expression in osteoclast precursor cells, supports their differentiation into osteoclasts by regulating the expression pattern of differentiation-related genes, especially those ones that are instrumental in cell fusion and bone matrix resorption. Moreover, the early, temporarily peaking activation of the ITGA2 gene brings it into a pole position in orchestrating the transcription of genes that are relevant in the later stage of osteoclastogenesis. Our study demonstrates that CD9 is an integrin α2β1-dependent gene. Despite the important roles of integrin α2β1 and CD9, we still observed syncytia formation in ITGA2-KO OCs, suggesting that other proteins besides CD9 and DC-STAMP contribute to osteoclastogenesis.

## Data Availability

The original contributions presented in the study are included in the article/Supplementary Material, further inquiries can be directed to the corresponding author.

## Abbreviations

DC-STAMP, dendritic cell-specific transmembrane protein; ECM, extracellular ma-trix; FcγR, Fc gamma receptor; GM-CSF, granulocyte-macrophage colony-stimulating factor; M-CSF, Macrophage-colony stimulating factor; OC, osteoclast; OSCAR, osteoclast associated receptor; RANK, receptor for activation of nuclear factor κB; RANKL, RANK ligand; ROI, region of interest; TEM, tetraspanin-enriched microdomain; TNT, tunneling nanotube; TRAP, tartrate-resistant alkaline phosphatase; TUNEL, Terminal deoxynucleotidyl transferase dUTP nick end labeling; WEKA, Waikato environment for knowledge analysis.
