# Supplementary material for: Integrin α2 is an early marker for osteoclast differentiation that contributes to key steps in osteoclastogenesis
Source: Front Cell Dev Biol. 2024 Aug 15;12:1448725. doi: 10.3389/fcell.2024.1448725 (PMC11363192; doi:10.3389/fcell.2024.1448725)
Supplement: Supplementary file 1 [file DataSheet1.pdf]

Supplementary Material

1 Supplementary Tables

Supplementary Table S1: qRT-PCR primer sequences

Gene target	Sequence (5'-3')	Product size (bp)	NCBI accession
GAPDH-forward	aaggtcatcccagagctgaa	138	NM_008084.4
-reverse	ctgcttcaccaccttcttga		
ITGA2-forward	tggtagttgtgaccgatggc	99	NM_008396.3
-reverse	ctgctatgccgaacctcagt		
ITGAV-forward	aggatgtttctcctggtggga	81	NM_008402.3
-reverse	acatttgagaacttgccctcct		
TRAP-forward	agagagaaagtcaagggagtgg	96	NM_001102405.1
-reverse	ggcctttgtcctcaatgttcc		
RANK-forward	gggtgattttcttttggtgggt	101	NM_009399.3
-reverse	ctccattatccaagaaccagtgc		
NFATc1-forward	accagctttccagtcccttc	131	NM_198429.2
-reverse	taactgtagtgttcttcctcggc		
DC-STAMP-forward	cggaacttagacacagggaga	141	NM_029422.4
-reverse	ggccacaaagcaacagactc		
OSCAR-forward	gtcctgtcgctgatactccag	94	NM_175632.3
-reverse	ctaggggcgctgttggtg		
CTSK-forward	acccttagtcttccgctcac	90	NM_007802.4
-reverse	cttgaacacccacatcctgc		
CD9-forward	cggttgtcgagtcccttctg	96	NM_007657.4
-reverse	gatgcacttgctacctcctttg		
MyoX-forward	cgaatgctatcgctgcttgtg	99	NM_019472.2
-reverse	aggatcagcttggtgctttcc		
M-Sec-forward	agcgcgcctttgatgagtt	107	NM_009396.2

-reverse	ctccacggaagtccagaagaac		

2 Supplementary Figures

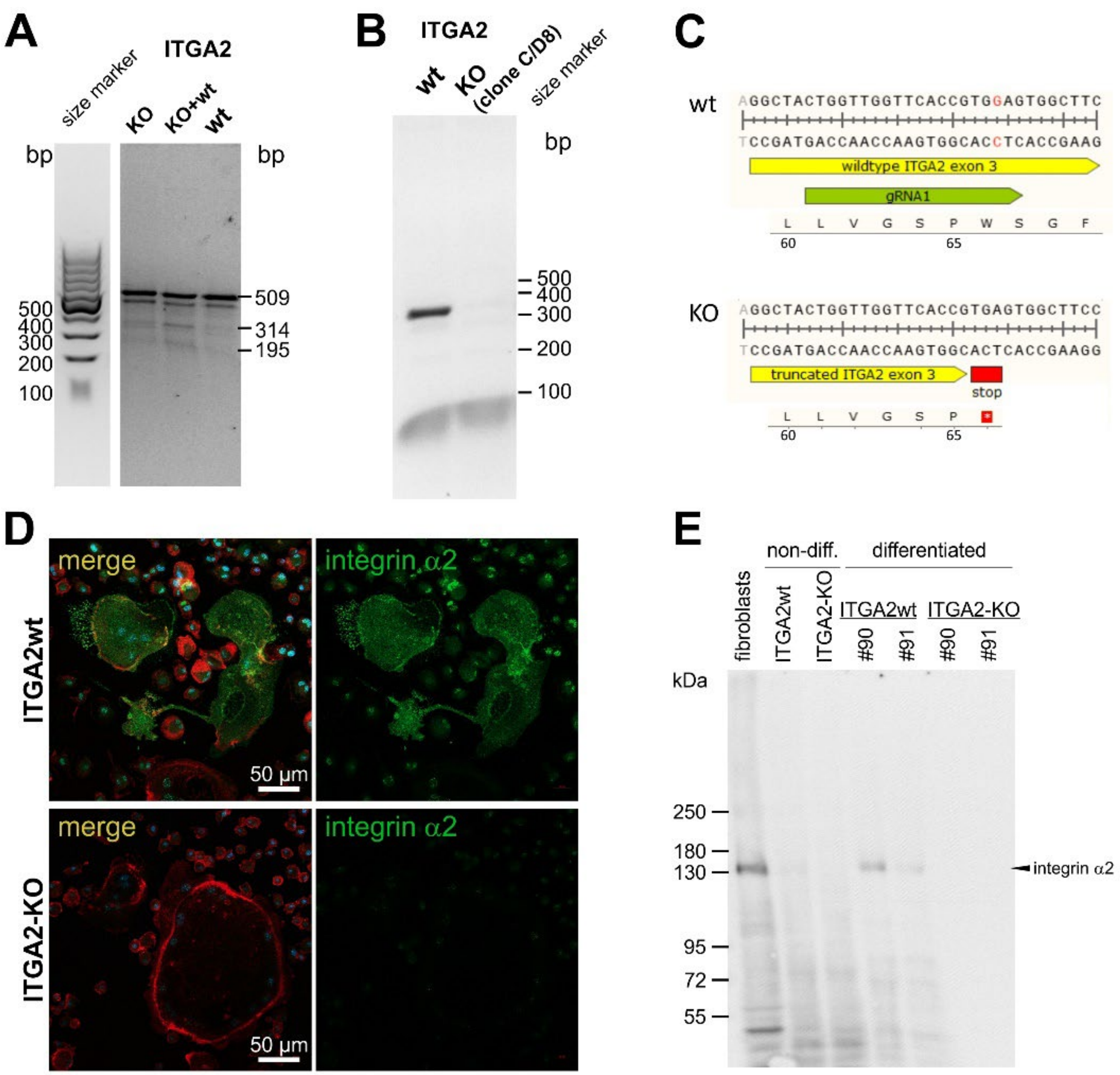

Supplementary Figure S1: Generation of ITGA2-knockout ER-Hoxb8 cells.

(**A**) Targeted knockout of the ITGA2 gene in ER-Hoxb8 cells was performed by lentiviral CRISPR/Cas9 transduction using a guide RNA homologous to the nucleotide sequence of the 3rd exon of mouse integrin α2 that encodes the amino acid sequence LLVGSP and introduces a nucleotide deletion in the following tryptophane codon. In T7-endonuclease test, a primer pair flanking the site homologous to the gRNA was used to amplify a nucleotide sequence that includes ITGA2 exon 3 by PCR. The PCR products were denatured and reannealed resulting in entirely matching fragments (509 bp) that cannot be cleaved by T7-endonuclease, or mismatching fragments. The latter are generated if a strand with an indel-mutation re-anneals with a wt strand, and are subsequently cleaved by T7-endonuclease to give rise to two additional DNA-fragments (314 and 195 bp) thereby revealing a successful indel mutation in the ITGA2 gene. The contrast of the image was enhanced for clarity. (**B**) PCR test of a clone picked from the ITGA2-KO transfection population

by using a primer corresponding to the guide RNA, thus allowing amplification only to the non-mutated ITGA2 gene sequence. (**C**) The ITGA2 gene was knocked out in ER-Hoxb8 cells using a lentivirally transduced guideRNA against ITGA2 exon 3 and CRISPR/Cas9 by deleting a single guanine base within the tryptophane codon (highlighted in red in the wildtype (wt) sequence) to generate a stop codon near the start of exon 3 in the knockout (KO) cells, as shown by sequencing. (**D**) Immunofluorescent staining of ER-Hoxb8 ITGA2wt- and ITGA2-KO cells at day 7 of differentiation on glass. F-Actin is depicted in red (phalloidin 647), integrin α2 in green, nuclei in blue. The right panels show the green fluorescence channelalone as a grayscale image. (**E**) Immunoblot analysis of integrin α2 expression in ER-Hoxb8 cells, either ITGA2wt or ITGA2-KO, in their non-differentiated form and after differentiation to osteoclasts for 4 days. Populations of ITGA2wt and ITGA2-KO cells, rather than individual clones, were grown under non-differentiating and differentiating conditions. Mouse fibroblasts served as ITGA2-expressing positive control. Cells were lysed and 50 µg of lysate proteins were separated by SDS-PAGE and afterwards blotted to a nitrocellulose membrane, which was stained with a rabbit anti-mouse integrin α2-antibody (NBP-67691, Novus Biological). Lysates from two independent differentiation experiments were tested and no integrin α2β1 was detectable in non-differentiated and in ITGA2 cell populations.

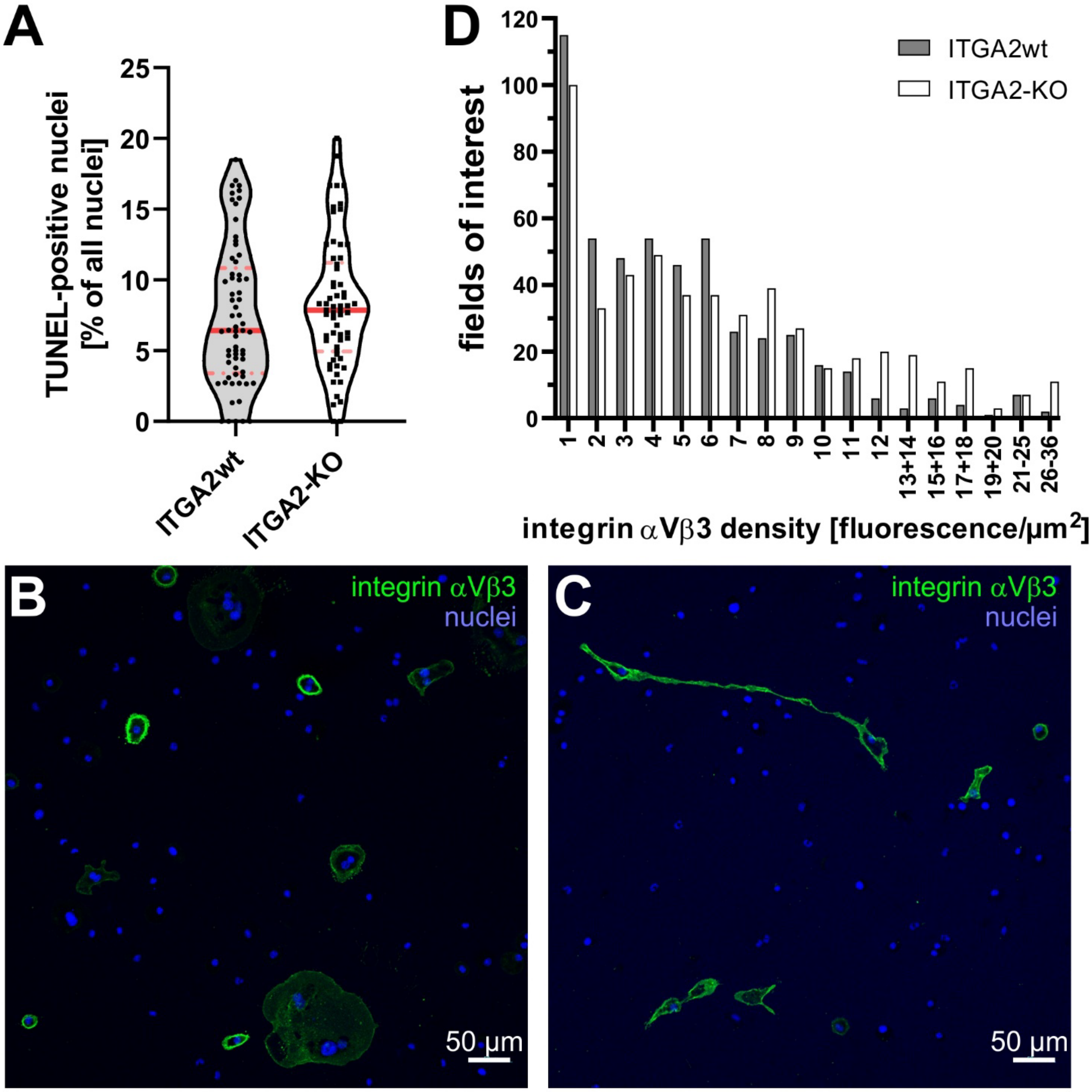

Supplementary Figure S2: ITGA2-KO cells differentiate into osteoclasts without increased premature apoptosis and with slightly elevated levels of integrin αV integrin.

(**A**) ER-Hoxb8 ITGA2wt and ITGA2-KO cells were induced for differentiation and fixed with 4% para-formaldehyde. With the terminal deoxynucleotidyl transferase dUTP nick end labeling (TUNEL) assay, apoptotic chromosome breaks were detected by enzymatic extension with Br-dUTP and subsequent incubation with a red fluorescent anti-BrdU-antibody. Cell nuclei were counterstained with 7-aminoactinomycin (7-AAD). Number of TUNEL-positive, apoptotic cells were counted and normalized to the overall number of nuclei to obtain the frequency of apoptotic cells, in each of the two populations. Each dot corresponds to the quantification of a field of view. Red lines indicate median (solid) and quartiles (dashed). (**B**) ITGA2wt and (**C**) ITGA2-KO cells differentiated for 6 days and stained for integrin αVβ3 with a rabbit-anti ITGAVB3 antiserum (Bioss bs-1310R) and a corresponding AlexaFluor 488-conjugated secondary antibody. The nuclei were stained in blue

with Hoechst dye. (**D**) Biometric quantification of the amount of αVβ3 integrin based on the cell area. Using at least 6 microscopic images of each population, as shown in (B) and (C), green fluorescence was quantified with Fiji (Image J 1.53t). Using its ROI manager, all green cells were delineated with a low threshold and the mean fluorescence intensity of each field of interest (cell, syncytia) was determined based on its area. The histogram bins more than 500 measurements for each cell population by fluorescence/area. The two frequency distributions obtained in this way differed significantly in a Chi^2 test ($p \leq 0.001$), indicating a slight compensatory upregulation of the αVβ3 integrin in some ITGA2-KO cells as compared to the wild type cells.

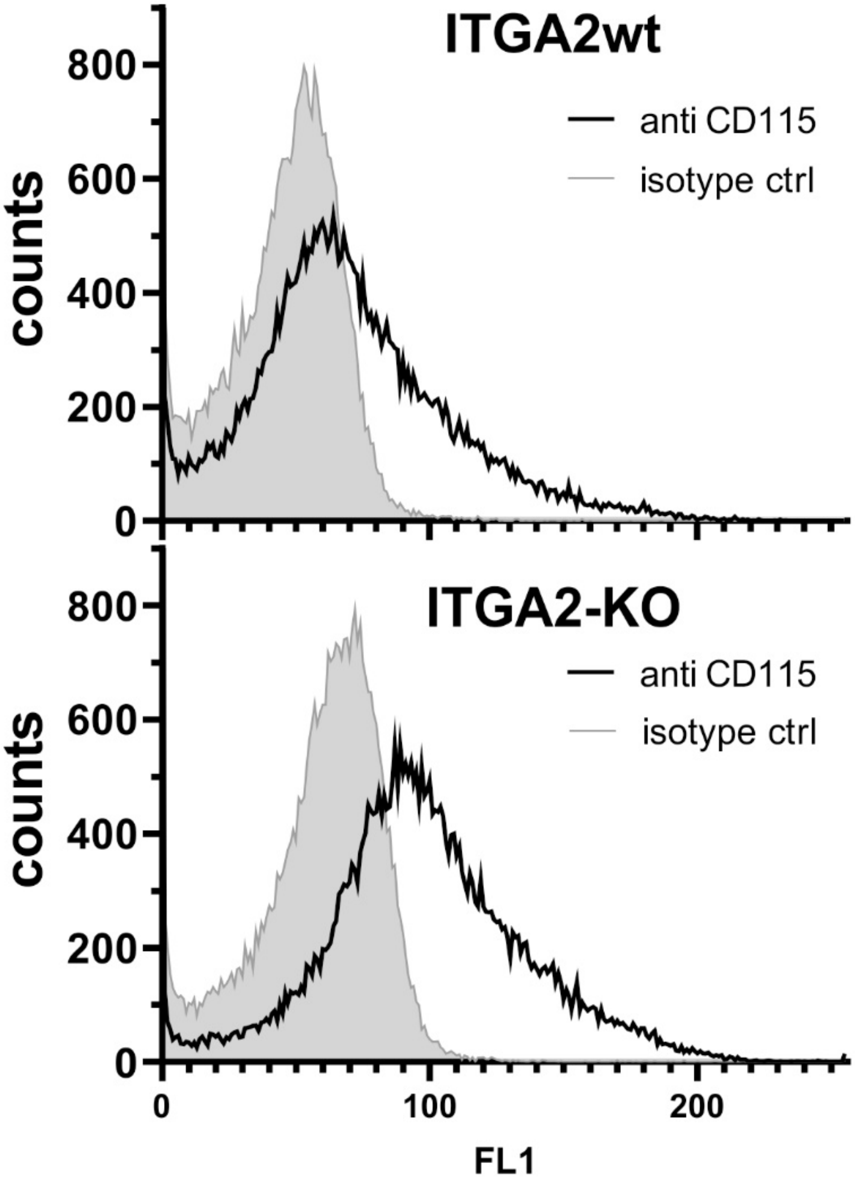

Supplementary Figure S3: M-CSF (CD115) expression is increased with ITGA2 loss.

ER-Hoxb8 wt and α2-knockout cells were sorted for the expression of the M-CSF receptor (CD115) using the CD115 MicroBead Kit from Miltenyi. Expression of CD115 was measured by flow cytometry in the CD115-enriched population of ITGA2-expressing and integrin-deficient ER-Hoxb8 cells.

3 Supplementary videos

This manuscript also contains two videos:

A: Video 1: ITGA2wt

B: Video 2: ITGA2-KO

Supplementary Videos: Real-time video microscopy of ER-Hoxb8 ITGA2-wt **(A)** and ER-Hoxb8 ITGA2-KO cells **(B)** during their osteoclastic differentiation on collagen-I coated glass coverslips at days 3.5- 5. ER-Hoxb8 ITGA2-KO cells differentiate somewhat slower than integrin α2 expressing cells and form more and longer, even branched protrusions. Many of these protrusions, so-called tunneling nanotubes, connect two or more cells, some of which fuse with each other in the further course of differentiation. Images were taken every 10min with the IncuCyte Live-Cell Analysis System at a magnification of 10× and subsequently assembled to time lapse videos. The timestamps are internal machine marks and do not represent the days of differentiation.
